# Multilocus Analysis of Divergence and Introgression in Sympatric and Allopatric Sibling Species of the *Lutzomyia longipalpis* Complex in Brazil

**DOI:** 10.1371/journal.pntd.0002495

**Published:** 2013-10-17

**Authors:** Alejandra S. Araki, Gabriel E. M. Ferreira, Camila J. Mazzoni, Nataly A. Souza, Ricardo C. Machado, Rafaela V. Bruno, Alexandre A. Peixoto

**Affiliations:** 1 Laboratório de Biologia Molecular de Insetos, Instituto Oswaldo Cruz, FIOCRUZ, Rio de Janeiro, Brazil; 2 Laboratório de Pesquisa em Leishmaniose, Instituto Oswaldo Cruz, FIOCRUZ, Rio de Janeiro, Brazil; 3 Institut für Zoo-und Wildtierforschung, Berlin, Germany; 4 Berlin Center for Genomics in Biodiversity Research, Berlin, Germany; 5 Laboratório de Transmissores de Leishmanioses, Instituto Oswaldo Cruz, FIOCRUZ, Rio de Janeiro, Brazil; 6 Instituto Nacional de Ciência e Tecnologia em Entomologia Molecular (INCT-EM), Rio de Janeiro, Brazil; The Faculty of Medicine, The Hebrew University of Jerusalem, Israel

## Abstract

**Background:**

*Lutzomyia longipalpis*, the main vector of visceral leishmaniasis in Latin America, is a complex of sibling species. In Brazil, a number of very closely related sibling species have been revealed by the analyses of copulation songs, sex pheromones and molecular markers. However, the level of divergence and gene flow between the sibling species remains unclear. Brazilian populations of this vector can be divided in two main groups: one producing Burst-type songs and the Cembrene-1 pheromone and a second more diverse group producing various Pulse song subtypes and different pheromones.

**Methodology/Principal Findings:**

We analyzed 21 nuclear loci in two pairs of Brazilian populations: two sympatric populations from the Sobral locality (1S and 2S) in northeastern Brazil and two allopatric populations from the Lapinha and Pancas localities in southeastern Brazil. Pancas and Sobral 2S are populations of the Burst/Cembrene-1 species while Lapinha and Sobral 1S are two putative incipient species producing the same pheromone and similar Pulse song subtypes. The multilocus analysis strongly suggests the occurrence of gene flow during the divergence between the sibling species, with different levels of introgression between loci. Moreover, this differential introgression is asymmetrical, with estimated gene flow being higher in the direction of the Burst/Cembrene-1 species.

**Conclusions/Significance:**

The results indicate that introgressive hybridization has been a crucial phenomenon in shaping the genome of the *L. longipalpis* complex. This has possible epidemiological implications and is particularly interesting considering the potential for increased introgression caused by man-made environmental changes and the current trend of leishmaniasis urbanization in Brazil.

## Author Summary

The sand fly *Lutzomyia longipalpis*, the most important vector of visceral leishmaniasis in the Americas, is a complex of cryptic species distributed from Argentina to Mexico. There is evidence for the existence of a number of closely related sibling species of this complex in

Brazil that differ in their male mating songs, sex pheromones and molecular markers. We compared the levels of divergence and gene-flow (introgression) in sympatric and allopatric pairs of sibling species of this complex using several molecular markers. Our results suggest that the *L. longipalpis* species complex in Brazil has an intricate evolutionary history with a crucial role for introgression, which varies between loci and is asymmetrical between the sibling species. This introgression potentially has important epidemiological implications because it could affect genes that influence the relative role that the different sibling species play as vectors. In addition, our results are particularly relevant considering the potential for increased introgression caused by man-made changes to the environment and the current trend of urbanization of visceral leishmaniasis in Brazil.

## Introduction

Speciation events are the result of a complex array of interesting and dynamic biological processes that remain only partially understood [1]. The intricate balance among mutation, recombination, gene flow, genetic drift and natural selection can either unify or differentiate genetic variation, and this consequently may or may not promote the appearance of new species [2]–[5]. The evolutionary history described by individual genes may be quite variable, and the pattern of relationships among closely related species can be discordant [6]–[8]. In principle, the use of multiple loci should give a more complete picture of the history of divergence of species complexes, and comparisons across genes can reveal whether all loci fit a simple model of dichotomic phylogeny. Use of multiple loci can also reveal whether retention of ancestral polymorphisms, introgression or selective pressures can explain incongruities in the evolutionary histories of species complexes [5], [8], [9].

Gene flow during a speciation process can be evidenced when some loci show little or no differentiation, while others show a large level. In the last decade, many studies have been carried out in species that have recently diverged, and it appears that divergence and speciation may often occur in the presence of gene flow [10]–[21]. This is also true for insect disease vectors, where the process of divergence with gene flow is particularly interesting beyond the standard evolutionary viewpoint. Genes involved in vectorial capacity, insecticide resistance and adaptation to different environmental conditions could be introgressing between sibling species with important epidemiological consequences [20], [22]–[26].

The most important neotropical vector of *Leishmania infantum*, the causative agent of American visceral leishmaniasis, is the sand fly *Lutzomyia longipalpis* (Diptera: Psychodidae), a complex of sibling species with a large distribution area ranging from northern Argentina and Uruguay to Mexico [27]–[36]. In Brazil, despite some incongruence among genetic markers [32], [34], an integrative analysis using a combination of biochemical, behavioral and molecular traits strongly supports a number of sibling species having different levels of differentiation [33].

The Brazilian populations of *L. longipalpis s.l.* can be divided in two main groups. The first group is genetically homogeneous and widely spread, and probably represents a single species. Males of this species produce Burst-type copulation songs and the Cembrene-1 pheromone. The second group is very heterogeneous and likely represents a number of putative incipient species with more restricted distributions. These sibling species produce different subtypes of Pulse-type copulation song in combination with different sex pheromones (Germacrene, Himachalene, Cembrene-1 and Cembrene-2) [33].

The coexistence of sibling species in an overlapping geographic area is one of the best pieces of evidence for the existence of a species complex [37], [38]; in at least three localities in Brazil, siblings of *L. longipalpis s.l.* are present in sympatry [33]. This is true for the Brazilian municipality of Sobral (Ceará State, Northeast Brazil), where two *L. longipalpis* sibling species were observed. In this locality, males of these two species can be distinguished by the number of pale spots on the abdomen (one or two pairs of spots: “Sobral 1S” and “Sobral 2S,” respectively). Crossing experiments show that these two siblings have strong reproductive isolation, which is consistent with the fact that their males produce different pheromones and copulation songs [29], [35], [39], [40]. In addition, molecular markers such as microsatellites and nuclear genes clearly indicate that Sobral 1S and Sobral 2S represent two sympatric species in Sobral [41]–[44]. However, some of these molecular markers also show signs of introgression [41], [43], and this could explain the differences in interpretation among early studies regarding the status of the Brazilian populations [32], [34].

In the current study, we conducted a multilocus approach using 21 nuclear loci to estimate and compare levels of divergence and gene flow between the sympatric siblings from Sobral and two allopatric species from the localities of Lapinha (Minas Gerais State) and Pancas (Espírito Santo State) in Southeast Brazil. Pancas and Sobral 2S are probably populations of the same species, whose males produce Burst-type songs and the Cembrene-1 pheromone, while Lapinha and Sobral 1S are two putative incipient species that share the same pheromone (Germacrene) and produce, respectively, Pulse song subtypes P2 and P3 [33]. In the absence of interbreeding, sympatric populations of two species should not be more similar to each other genetically than to allopatric populations of these two species. This kind of comparison using a larger number of unlinked loci allows to distinguish among common ancestry and the effects of introgression. Moreover, it brings new insights of how this is shaping the genome of the *L. longipalpis* species complex.

## Methods

**Figure 1:**
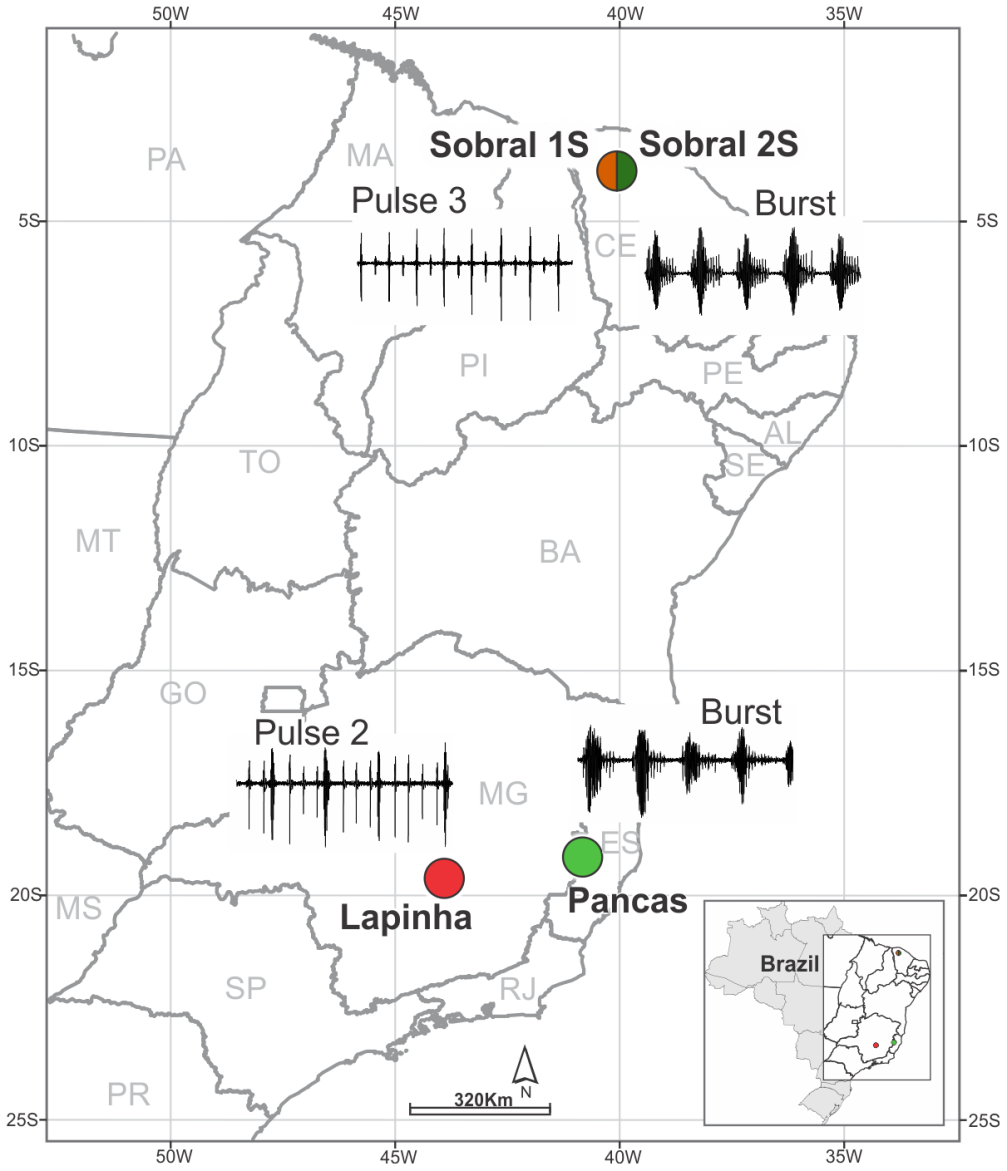
Geographic locations of the Brazilian populations of *Lutzomyia longipalpis* species complex studied. The circles show the approximate location of the two sympatric sibling species from Sobral (Ceará State) and the two allopatric sibling species from Pancas (Espírito Santo State) and Lapinha (Minas Gerais State). Divided circle represents the two sympatric species Sobral 2S (green) and Sobral 1S (orange), with Burst and Pulse (subtype 3) types of copulation songs, respectively. Circles in light green and red correspond to the allopatric species Pancas and Lapinha that produce Burst and Pulse (subtype 2) types of copulation songs, respectively.

Sand flies were collected from Sobral (Ceará state) (3°41′S, 40°20′W) in northeastern Brazil, Pancas (Espírito Santo state) (19°13′S, 40°51′W) and Lapinha (Lagoa Santa, Minas Gerais state) (19°38′S, 43°53′W) in southeastern Brazil (Figure 1). A permit for sand fly collection in Brazil was obtained from the Brazilian Ministry of Environment (SISBIO #26066-1). Sand flies were captured using CDC light-traps near human habitation with permission from local homeowners. In addition, the collections were usually supported by the local vector surveillance authorities from local State Health Departments. Samples were identified according to [45], and only males were used to avoid misidentification, as females are difficult to distinguish from other closely related species.

Twenty-one loci were used in the analysis (Table S1). Three of these loci, *period* (*per*) (accession numbers AY082911–AY082957, AF446186–AF446164, EU713179–EU713153), *cacophony* (*cac*) (accession numbers AF493078–AF493142, AF528965–AF529009, AF493120–AF493101) and *paralytic* (*para*), were used in previous studies of the *L. longipalpis* complex (accession numbers EU746318–EU746365, JQ359112–JQ359114, JQ359116, JQ359118, JQ359120, JQ359122, JQ359124–JQ359134, JQ359136, JQ359138–JQ359139, JQ359264–JQ359266, JQ359268–JQ359272, JQ359274–JQ359276, JQ359278–JQ359295) [33], [36], [42]–[44], [46].

The remaining 18 loci (Table S2) are new markers randomly selected from a screen of cDNA sequences performed in our lab. The selected loci are responsible for different functions (Table S1), show a high similarity at the protein level when compared to *Anopheles gambiae* and/or *Drosophila melanogaster* and contain one intron in the latter species under the assumption that they might also be present in the *L. longipalpis* studied fragment, although that was not always the case.

DNA samples used for PCR were prepared as previously described [24]. PCR was carried out using proofreading *Pfu* DNA Polymerase (Biotools) for 35 cycles: 95°C for 30 s, 55°C for 30 s, and 72°C for 30 s. PCR products were purified using the Wizard PCR Preps kit (Promega) or Micro Spin S-400 HR (GE Healthcare) and were cloned into the pMOS Blunt-ended Vector (GE Healthcare). The plasmid DNA was prepared using the alkaline lysis method in 96 well micro-plates [47] and filtered in multiscreen filter plates. Sequencing was carried out using the ABI Prism Big Dye Terminator Cycle Sequencing Ready Reaction V3.1 kit (Applied Biosystems) in an ABI 3730 DNA Sequencer by the PDTIS (FIOCRUZ, Rio de Janeiro, Brazil) sequencing service [48]. The sequences have been submitted to GenBank (accession numbers JX301771–JX303202).

The DNA sequences were edited and aligned using Bioedit v.7.0.9.0 [49] or MEGA5 [50]. The polymorphism summaries, the neutrality tests and the differentiation among populations were estimated using DnaSP5 [51] and ProSeq 2.91 software [52]. The HKA multilocus test [53] of neutral molecular evolution was carried out using HKA software (http://genfaculty.rutgers.edu/hey/home). Haplotype networks were constructed using statistical parsimony [54] implemented in the TCS software [55].

We analyzed our data set under the isolation-with-migration model available with the IM software [12] to discriminate between the relative effects of divergence and gene flow. This multilocus analysis was performed using non-recombining blocks (NRB) of the 21 different nuclear loci. Sites within indels or ambiguous alignment were removed (Table S3). To select the NRB, we used IMgc software [56] for the Sobral 1S and 2S sequences and manually selected the blocks and sequences of Pancas and Lapinha according to the NRB from Sobral to ensure consistency. Some putative recombinant sequences were excluded from the data set. The IM model considers parameters for splitting time (*t*), bidirectional gene flow after splitting (*m_1_* and *m_2_*) and current effective population sizes (*θ_1_*, *θ_2_* and *θ_A_*). To fit the model to our data, the IM software used a Bayesian framework that gave estimates for the posterior probability densities of the model parameters using a Markov chain Monte Carlo simulation [12]. We carried out two sets of analyses, one comparison involving the sympatric siblings from Sobral (Sobral 1S *vs.* Sobral 2S) and one for the two allopatric siblings (Pancas *vs.* Lapinha). For each analysis, we ran simulations assuming the Infinite Site model (IS) [57] of sequence evolution recommended for nuclear loci. In addition, for each comparison (sympatric or allopatric) we ran simulations that estimated the population migration rates and simulations that estimated the per locus migration rates. We conducted preliminary runs to determine appropriate priors for subsequent runs. After that we performed three to five independent runs with different starting points to ensure that the values converged on similar estimates, with ten or twenty independent chains under Metropolis coupling. Each chain was initiated with a burn-in period of 200,000 updates, and the total run length of each analysis was between 30 million and 40 million updates, using the following input parameters for simulations: *m* = 25 and 100, *θ* = 5 and 10, *t = *5. Convergence was assessed by estimating the effective sample size, which was always over 50. The six demographic parameters were calculated from the values of the bin with the highest count; these values corresponded to the average calculated from three to five independent runs. For credibility intervals for each parameter, we recorded the 90% posterior density interval, the shortest span that includes 90% of the probability density of a parameter. To convert the parameter estimates into demographic values, we used *Drosophila melanogaster* synonymous and non-synonymous substitution rates for nuclear genes, 1.56×10^−8^ and 1.91×10^−9^ per site per year, respectively [58]. From the 21 loci used in this study, we calculated a geometric mean of mutation rate per loci per year, μ = 1.77×10^−6^, that was used to rescale the IM parameter estimates. This value was used to convert the parameter estimate *t* to the number of years since population splitting (t), assuming that *L. longipalpis s.l.* has about ten generations per year, and to convert the estimates of the population mutation rates (*θ_1_*, *θ_2_* and *θ_A_*) into estimates of the effective population sizes (*N_1_*, *N_2_* and *N_A_*).

## Results

### Intra-population nucleotide variation

Our data set included 21 loci. The markers are responsible for different functions and potentially spread throughout the genome of *L. longipalpis*, based on their positions in *A. gambiae* and *D. melanogaster* (Table S1, see also Methods for more details).

Table 1 shows a summary of the multilocus DNA polymorphisms for the four *L. longipalpis s.l.* populations sampled in eastern Brazil (Figure 1). In general, for each molecular marker, the levels of variation within populations were similar. However, we observed considerable variation in the levels among loci. The *CG9297*, *rpL36*, *tfIIAL* and *obp19a* loci were the most polymorphic, and *sesB*, *eno*, *tropC* and *CG9769* were the least polymorphic of the markers analyzed. Indels were observed in thirteen markers, and all were located in introns.

**Table 1:** Polymorphism summary for the 21 loci in four siblings of *L. longipalpis* complex from Brazil.

		N				S				π				Θ			
Locus	Length (bp)^1^	S1S	S2S	Lap	Pan	S1S	S2S	Lap	Pan	S1S	S2S	Lap	Pan	S1S	S2S	Lap	Pan
*CG9297*	474	32	29	18	25	36	37	42	32	0.024	0.019	0.028	0.009	0.019	0.020	0.027	0.019
*CG9769*	378	24	24	20	14	12	8	1	3	0.005	0.035	0.000	0.002	0.009	0.006	0.001	0.003
*eno*	245	28	32	29	30	4	9	3	5	0.002	0.003	0.003	0.002	0.004	0.009	0.003	0.005
*kinC*	664	17	23	24	24	25	28	32	31	0.010	0.009	0.012	0.010	0.011	0.012	0.013	0.013
*mlcc*	217	30	28	17	24	12	10	7	7	0.005	0.006	0.008	0.007	0.012	0.014	0.010	0.009
*norpA*	104	32	30	38	25	8	5	9	9	0.020	0.014	0.017	0.024	0.019	0.012	0.021	0.023
*obp19a*	164	32	31	28	26	16	17	13	13	0.029	0.033	0.026	0.028	0.024	0.026	0.020	0.021
*rpL17A*	302	31	31	25	14	13	22	7	2	0.013	0.014	0.007	0.003	0.011	0.019	0.006	0.002
*rpL36*	450	21	16	32	23	44	48	25	26	0.025	0.031	0.020	0.015	0.029	0.034	0.015	0.017
*rpS19*	300	33	40	27	10	20	27	17	10	0.016	0.019	0.017	0.010	0.017	0.021	0.015	0.012
*sesB*	89	23	26	21	15	2	1	0	2	0.004	0.006	0.000	0.004	0.006	0.003	0.000	0.007
*slh*	258	27	30	19	16	22	24	11	12	0.028	0.018	0.011	0.015	0.023	0.024	0.013	0.015
*sec22*	429	17	15	29	16	26	19	14	24	0.013	0.015	0.007	0.020	0.018	0.014	0.008	0.017
*sod2*	310	26	27	27	16	10	15	5	3	0.008	0.008	0.006	0.002	0.009	0.013	0.005	0.003
*tfIIAL*	377	14	18	20	38	23	33	29	28	0.020	0.030	0.020	0.020	0.022	0.029	0.025	0.019
*tropC*	483	27	27	29	31	12	12	3	10	0.003	0.004	0.000	0.003	0.007	0.007	0.002	0.005
*up*	404	21	22	14	16	19	11	14	10	0.010	0.006	0.011	0.009	0.013	0.008	0.011	0.008
*ζcop*	386	30	16	28	21	14	10	6	7	0.005	0.007	0.003	0.004	0.010	0.008	0.004	0.005
*cac*	109	23	22	20	26	12	5	14	6	0.025	0.025	0.066	0.026	0.043	0.018	0.053	0.021
*para*	377	27	22	21	29	12	8	2	10	0.004	0.004	0.002	0.005	0.009	0.006	0.002	0.007
*per*	266	24	23	23	19	27	22	20	18	0.024	0.017	0.018	0.014	0.027	0.022	0.020	0.019

1 Aligned sequence length in base-pairs; N, number of sequences; S, segregating sites; π, nucleotide divergence average number of pair-wise differences; Θ, nucleotide divergence based on the number of segregating sites. S1S, Sobral one spot; S2S, Sobral two spot; Lap, Lapinha; Pan, Pancas.

Table 2 shows the Tajima's *D* statistics [59]. Although not significantly different from zero after Bonferroni's correction, values were negative in most cases, possibly indicating population expansion. Additional tests, such as the Fu's *F*_s_ [60] and Ramos-Onsins and Rozas *R* ^2^ [61] (considered more powerful to detect population growth [62]) also support the expansion hypothesis, mainly in Sobral (Table S4). Nevertheless, the HKA multilocus test showed no significant deviations from the neutral expectations in the sympatric pair (X^2^_Sobral 1S–Sobral 2S_ = 9.26, df = 40, P>0.99) or in the allopatric pair of species comparisons (X^2^_Lapinha–Pancas_ = 26.15, df = 40, P>0.95), indicating no obvious departures from neutrality in either case.

**Table 2:** Tajima's neutrality test of 21 loci for the four siblings of *L. longipalpis* from Brazil.

	Tajima's *D*			
Locus	S1S	S2S	Lap	Pan
*CG9297*	0.484	−0.708	0.676	−1.970
*CG9769*	−1.620	−1.244	−1.164	−0.886
*eno*	−1.721	−1.977	0.075	−1.749
*kinC*	−0.511	−0.970	−0.613	−0.974
*mlcc*	−1.710	−1.998	−0.461	−0.449
*norpA*	−0.319	−0.112	−0.510	−0.602
*obp19a*	0.196	0.192	0.603	1.122
*rpL17A*	0.211	−1.072	0.155	0.985
*rpL36*	−0.753	−0.598	1.257	−0.247
*rpS19*	−0.447	−0.600	0.287	−0.782
*sesB*	−0.636	1.591	-	−1.002
*slh*	0.550	−1.230	−0.639	−0.035
*sec22*	−1.219	−0.884	0.118	0.118
*sod2*	−0.279	−1.270	0.645	−0.525
*tfIIAL*	−0.742	0.012	−0.736	0.158
*tropC*	−1.823	−1.371	−1.733	−1.507
*up*	−1.082	−0.988	0.045	0.486
*ζcop*	−1.451	−0.929	−0.786	−0.608
*cac*	−1.484	0.726	0.176	−1.970
*para*	−1.878	−1.081	0.222	−0.832
*per*	−0.538	−0.852	−0.432	−1.007

S1S, Sobral 1S; S2S, Sobral 2S; Lap, Lapinha; Pan, Pancas.

### Differentiation and introgression between sympatric and allopatric pairs of sibling species

**Figure 2:**
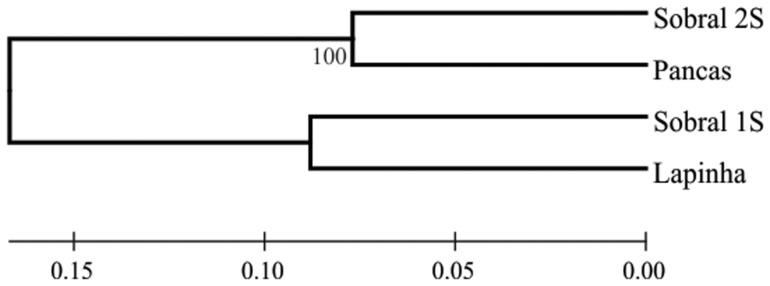
Neighbor joining tree of *Lutzomyia longipalpis* populations based on the pairwise *F*_ST_ of 21 loci. The bootstrap value is based on 100 replicates in which the 21 loci were bootstrapped.

We carried out a comparison of the differentiation between the two pairs of sibling species: sympatric (Sobral 1S *vs.* Sobral 2S) and allopatric (Lapinha *vs.* Pancas). As mentioned before, Pancas and Sobral 2S are populations of the Burst/Cembrene species while Lapinha and Sobral 1S are two putative incipient species producing the same pheromone (Germacrene) and Pulse song subtypes P2 and P3 [33]. Table 3 shows the *F*_ST_ values for each locus for the Sobral 1S *vs.* Sobral 2S and Lapinha *vs.* Pancas comparisons. Table S5 shows the values for the other pairwise comparisons while Table S6 exhibits Nei's genetic distance (Dxy) for all pairwise comparisons. A tree of these four populations based on the mean *F*_ST_ values clearly shows that the closer genetic similarity of the two Burst-type populations (Pancas and Sobral 2S) at one side and the two Pulse-type populations (Lapinha and Sobral 1S) in the other (Figure 2). In addition, it also shows that although Lapinha and Sobral 1S produce different subtypes of Pulse songs and probably represent incipient species, the overall level of genetic divergence between them is only slightly higher than between the two Burst-type populations.

**Table 3:** Differentiation between sympatric and allopatric siblings of *L. longipalpis* complex from Brazil.

	Sympatric species			Allopatric species		
	Sobral 1S *vs* Sobral 2S			Lapinha *vs* Pancas		
Locus	*F*_ST_	*Ss*	*Sf*	*F*_ST_	*Ss*	*Sf*
*CG9297*	0.106***	26	0	0.486***	9	0
*CG9769*	0.059*	3	0	0.954***	0	8
*eno*	0.007^ns^	2	0	0.134***	0	0
*kinC*	0.145**	10	0	0.331***	10	0
*mlcc*	0.021^ns^	3	0	0.250***	0	0
*norpA*	0.000^ns^	6	0	0.130***	3	0
*obp19a*	0.032*	14	0	0.138***	8	0
*rpL17A*	0.115***	11	0	0.782***	0	2
*rpL36*	0.034^ns^	29	0	0.300***	9	0
*rpS19*	0.351***	10	0	0.486***	3	0
*sesB*	0.334***	0	0	0.796***	0	0
*slh*	0.184***	13	0	0.568***	0	0
*sec22*	0.475***	3	0	0.461***	1	1
*sod2*	0.276***	7	0	0.337***	0	0
*tfIIAL*	0.165***	15	0	0.350***	20	0
*tropC*	0.359***	5	0	0.705***	0	1
*up*	0.547***	5	0	0.438***	0	1
*ζcop*	0.036^ns^	5	0	0.454***	0	0
*cac*	0.076*	4	0	0.130***	4	0
*para*	0.766***	1	3	0.815***	0	4
*per*	0.395***	6	0	0.471***	3	0

*F*_ST_, pairwise fixation index. Significance evaluated with 1000 permutations;

*** , significant at P<0.001;

** , significant at P<0.01;

* , significant at P<0.05;

ns , non-significant P>0.05.

*Ss*, shared sites; *Sf*, fixed sites.

**Figure 3:**
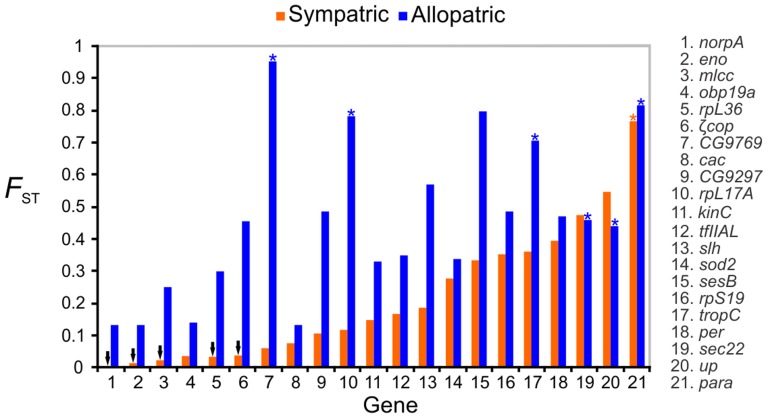
The distribution of *F*_ST_ values comparing sympatric and allopatric *Lutzomyia longipalpis* species from Brazil. Bars indicate the *F*_ST_ values for 21 nuclear markers from sympatric (orange) and allopatric (blue) pairs of species. The *F*_ST_ values were ordered according to ascending values from the sympatric comparisons. Black arrows indicate non-statistically significant values, and asterisks show markers with fixed polymorphic sites.

The sympatric pair shared polymorphisms in almost all loci and only exhibited fixed differences in *para* (Table 3). On the other hand, the allopatric pair of species only shared polymorphisms in about half of the 21 loci and a total of 17 fixed differences were observed in six loci. The lower level of divergence between sympatric species than allopatric species was also observed after inspection of the observed *F*_ST_ values (Table 3 and Figure 3). The mean pairwise *F*_ST_ was more than twice as high for the allopatric pair (0.453±0.245) than for the sympatric pair (0.213±0.209), and this difference was significant (t = −3.413; p<0.01). While all 21 *F*_ST_ values were significant in the case of the allopatric pair, five of the values for the sympatric siblings failed to reach significance. Out of 21 loci, only two (*sec22* and *up*) showed higher *F*_ST_ values between the sympatric pair than between the allopatric populations.

**Figure 4:**
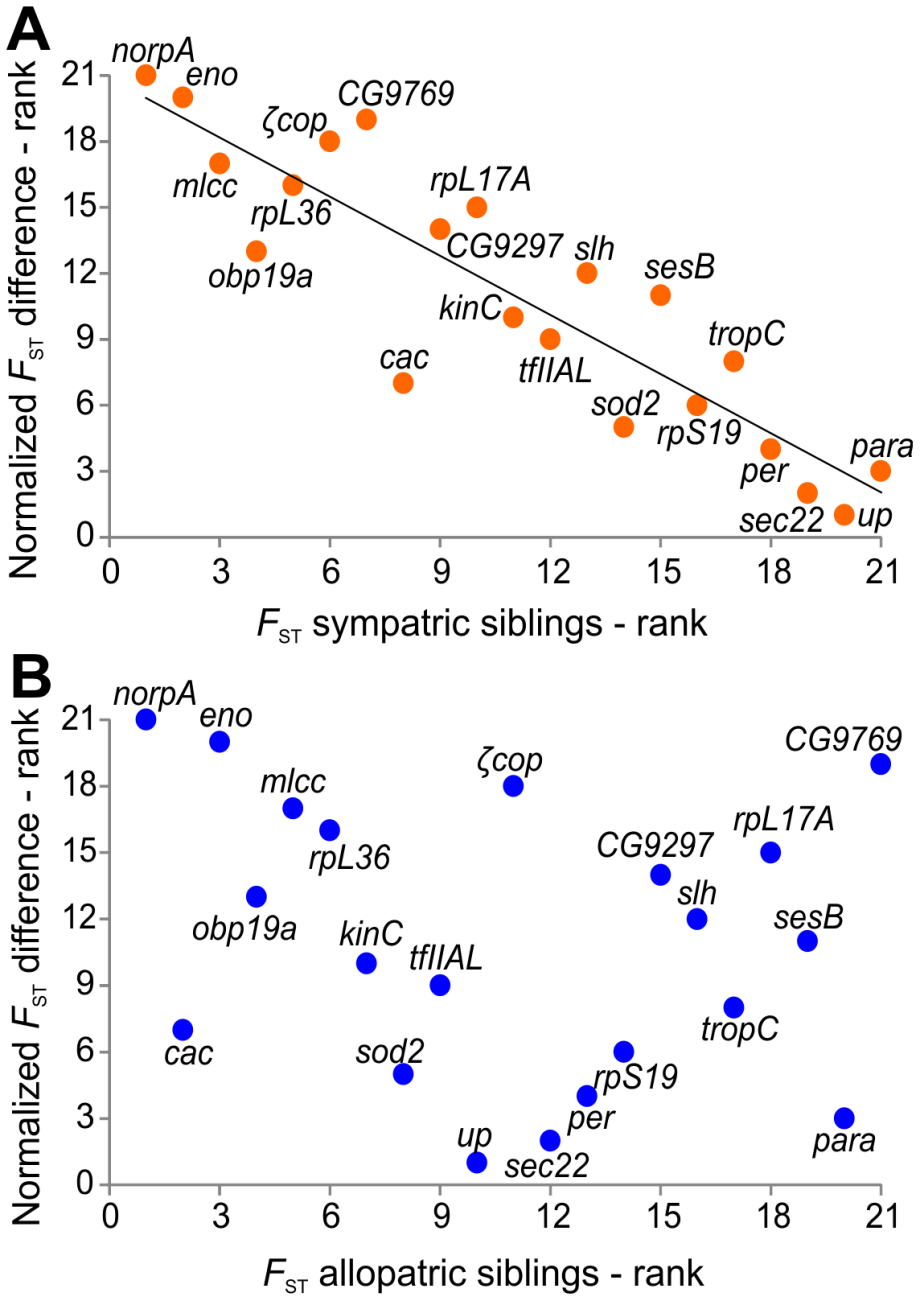
Correlation between species' *F*_ST_ value ranks and the normalized *F*_ST_ differences ranks. (A) Correlation between the ranks of the normalized differences in *F*_ST_ values [(*F*_ST_ Allopatric−*F*_ST_ sympatric)/*F*_ST_ allopatric] and the ranks of *F*_ST_ values for the sympatric species pair at 21 markers. High significant negative correlation was observed, and the trend line for all data calculated by ordinary least squares regression is shown by a solid line. (B) Correlation between the ranks of the normalized differences in *F*_ST_ and the ranks of *F*_ST_ values for the allopatric species pair at 21 markers.

The data in Table 3 and Figure 3 strongly suggest the occurrence of introgression between the sympatric populations. In addition, the estimated *F*_ST_ statistics were highly variable between loci in both comparisons. Part of the variation was likely caused by differences in the rate of evolution of the different loci. However, as shown in Figure 3, differences in *F*_ST_ values between the allopatric and sympatric comparisons tend to be lower for loci that have high *F*_ST_ values in the sympatric pair. In fact, Figure 4A shows that there is a highly significant negative correlation (r = −0.896; p<0.001) between the ranked (from the smallest to the highest) normalized differences in *F*_ST_ values between the sympatric and allopatric pairs at each loci and the respective rank of *F*_ST_ values in the sympatric pair at the same loci (see figure legend for more details). No correlation is observed in the case of the allopatric populations (r = 0.231; p = 0.3137) (Figure 4B). These results suggest that selection is acting as a filter on gene flow and, as a result, introgression is differential, affecting some loci to a much greater extent than others. This shows that, while one can predict that a molecular marker with a high *F*_ST_ value in a sympatric comparison will most likely show a similar value between two allopatric sibling species, the same is not true for many of the high *F*_ST_ values observed between allopatric populations. A gene that differentiates sympatric species is likely a good general marker for the complex. The same is not necessarily true for markers that differentiate allopatric species.

**Figure 5:**
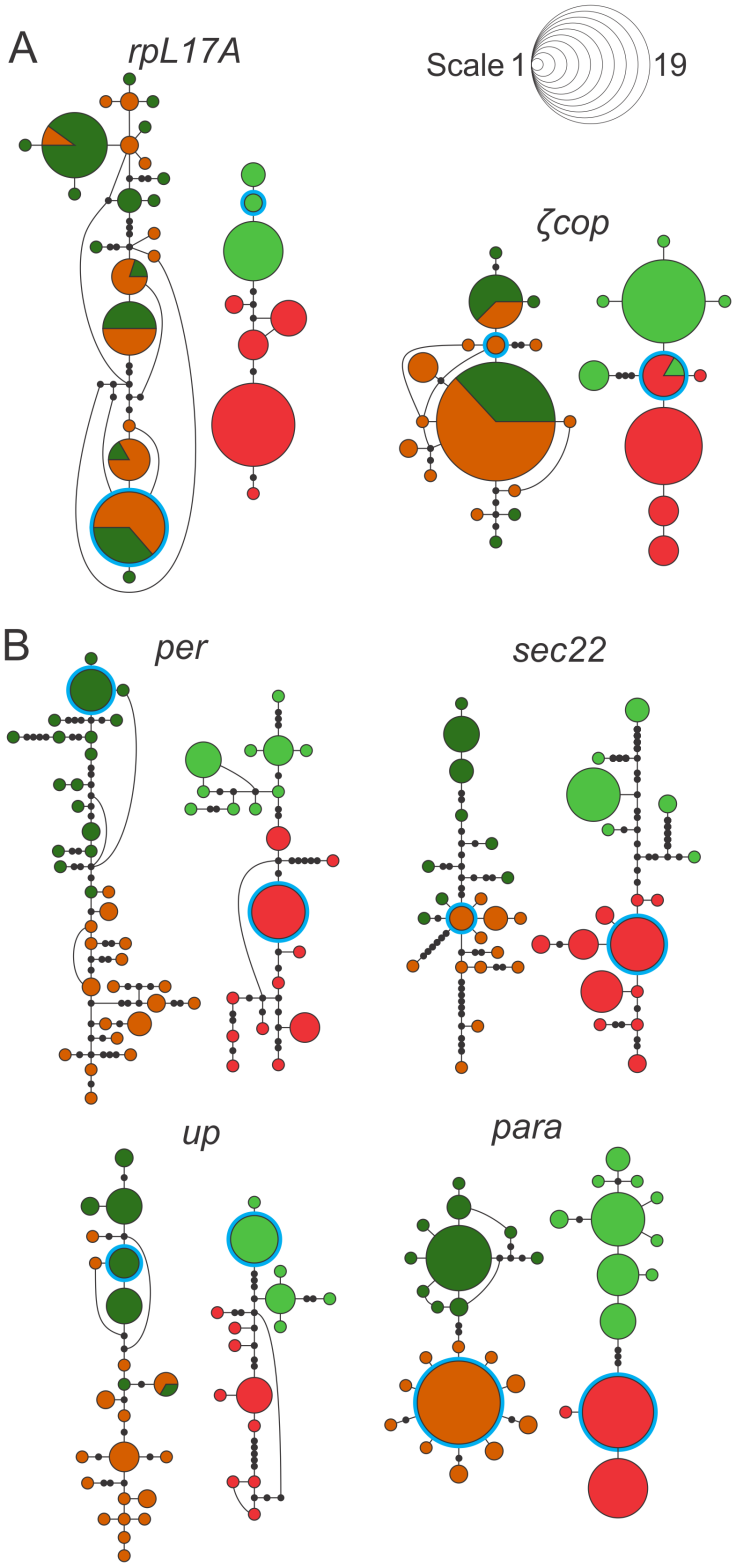
Haplotype networks from sympatric and allopatric comparisons of selected loci with extreme *F*_ST_ values. (A) The network of the loci *ζcop* and *rpL17A* from the sympatric species of the *Lutzomyia longipalpis* complex shows mixed a haplotype distribution, unlike the two well-separated cluster for the allopatric species. This is in agreement with the low degree of divergence between sympatric and high divergence between allopatric species. (B) The loci *per*, *sec22*, *up* and *para* presented sympatric and allopatric networks with haplotypes separated by species group, which also corroborates the high values of pairwise *F*_ST_ (see Table 2). Each *L. longipalpis* population is represented by one color: Sobral 1S (orange), Sobral 2S (green), Lapinha (red) and Pancas (light green). Colored circles represent unique haplotypes, and their sizes are proportional to their frequencies. Black circles are hypothetical haplotypes. Curved lines represent alternative branching between haplotypes.

Figure 5 illustrates this further by comparing the haplotype networks of a few markers (*ζcop*, *rpL17A*, *per*, *up*, *sec22* and *para*) in sympatric and allopatric populations. The first two, *rpL17A* and *ζcop* (Figure 5A), represent examples of markers which showed high *F*_ST_ values in allopatry but low values in sympatry (Figure 3). The allopatric networks of these two loci show much better separation between the haplotypes, especially in the case of *rpL17A*, which shows no separation at all in sympatry. On the other hand, in the case of the four loci with high *F*_ST_ values in sympatry (*per*, *up*, *sec22* and *para*), the networks are somewhat similar in the allopatric and sympatric comparisons (Figure 5B). Therefore, these markers seem to be more reliable for the study of the divergence between populations of the *L. longipalpis* complex as they avoid the problems caused by strong introgression.

The results presented above show a complex pattern of divergence and gene flow between the Brazilian species of the *L. longipalpis* complex. In particular, we found evidence of differential introgression among loci in the sympatric pair of species. To explore in more detail the gene flow between sympatric and allopatric relatives, we performed a multilocus analysis using the IM software.

### Isolation with migration analysis

Because the IM software requires the absence of recombination within loci, a non-recombining block (NRB) was extracted from each gene alignment, and some putative recombinant sequences were excluded from the data set (Table S3, see Methods). To evaluate the effects of the decrease in fragment length and/or the number of sequences per loci, we conducted polymorphism and divergence analyses (Table S7 and S8) similar to that carried out for the whole fragment (WF). The within-population variation in NRB was not significantly different from WF in any of the populations (Wilcoxon test: π_Sobral 1S_: p≥0.782; θ_Sobral 1S_: p≥0.890; π_Sobral 2S_: p≥0.818; θ_Sobral 2S_: p≥0.495; π_Lapinha_: p≥0.891; θ_Lapinha_: p≥0.546; π_Pancas_: p≥0.323; θ_Pancas_: p≥0.312). In addition, the comparisons between pairs of *F*_ST_ values also showed that the differences between NRB and WF remained small and unbiased in most cases (Wilcoxon test: *F*_ST_ _Sobral 1S *vs.* Sobral 2S_: p≥0.159 and *F*_ST_ _Lapinha *vs.* Pancas_: p≥0.444).

**Figure 6:**
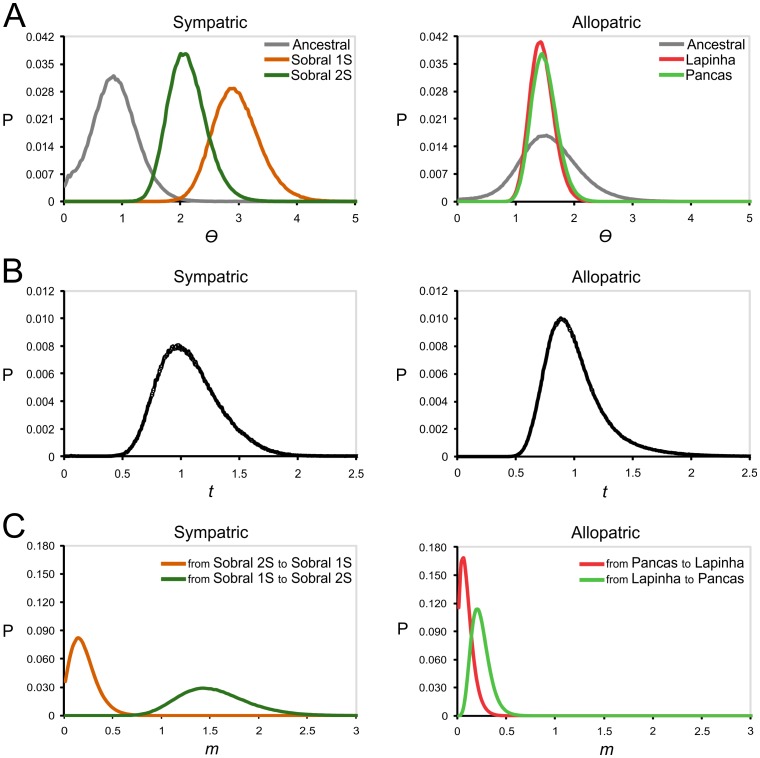
Distributions of marginal posterior probabilities of model parameters of sympatric and allopatric species. Probability densities (P) are shown in curves, which present single narrow peaks for six parameters of the isolation with migration model: (A) theta (*Θ* = 4Nμ), (B) divergence time between species (*t* = tμ) and (C) migration rate (*m* = m/μ).

IM parameters (Table 4) were estimated to infer and compare the population history of the two sympatric and the two allopatric sibling species of the *L. longipalpis* complex. Figure 6 shows the posterior probability distributions for six parameters with single narrow peaks and bounds that fell within the prior distributions. In all cases, each of the replicates yielded a posterior distribution with identical and well-defined modes. The marginal posterior distribution for *θ* of the sympatric species showed slightly different distributions (Figure 6A). The current effective population sizes estimated from these values indicate a three-fold increase for Sobral 1S and a two-fold increase for Sobral 2S since their splitting from the ancestral population. On the other hand, the *θ* parameters for Pancas and Lapinha indicate that these two allopatric relatives and the ancestral population have similar effective population sizes (Figure 6A). The marginal posterior distributions for *t* suggest that, as expected, the sympatric pair of species split from the ancestral population at approximately the same time as the allopatric pair (Figure 6B). Therefore, assuming mutation rates similar to *Drosophila* (see Material and Methods), the splitting event that separated the Burst-type (Sobral 2S and Pancas) and Pulse-type song (Sobral 1S and Lapinha) lineages occurred approximately 0.5 MYA (Table 4).

**Table 4:** Estimates of parameters from IM analysis of sympatric and allopatric comparisons.

Comparison	Estimates of parameters					
Sympatric	*θ*_S1S_	2.9127	(2.2693–3.6479)	N_S1S_	4,094,250	(3,189,942–5,127,705)
	*θ*_S2S_	2.056	(1.5213–2.6660)	N_S2S_	2,890,075	(2,138,459–3,747,539)
	*θ_A_*	0.8661	(0.2109–1.5138)	N_A_	1,217,493	(296,387–2,127,881)
	*t*	0.9463	(0.6388–1.4613)	t	532,049	(359,151–821,619)
	*m*_S1S_	0.1375	(0.9125–2.0938)	2Nm	0.2002	(0.0142–0.7524)
	*m*_S2S_	1.425	(0.0125–0.4125)	2Nm	1.4649	(0.6941–2.7910)
Allopatric	*θ*_Pan_	1.4355	(1.0803–1.8506)	N_Pan_	2,017,852	(1,518,555–2,601,349)
	*θ*_Lap_	1.4134	(1.0549–1.7923)	N_Lap_	1,986,716	(1,482,851–2,519,328)
	*θ_A_*	1.4901	(0.6760–2.4426)	N_A_	2,094,637	(950,169–3,433,476)
	*t*	0.8863	(0.6138–1.3863)	t	498,313	(345,094–779,449)
	*m*_Pan_	0.2125	(0.0625–0.4375)	2Nm	0.1525	(0.0338–0.4048)
	*m*_Lap_	0.0625	(0.0125–0.2625)	2Nm	0.0442	(0.0066–0.2352)

*θ*, the estimated population mutation rates; *t*, the estimated number of mutations since the time of splitting; *m*, the estimated migration rates; N, the estimated effective population sizes; t, the estimated time in years at which the ancestral population split into the sampled populations; 2Nm, the estimated effective number of migrant gene copies per generation in each direction. The 90% lowest and highest posterior density intervals for each parameter are shown in brackets. _S1S_, Sobral 1S; _S2S_, Sobral 2S; _Pan_, Pancas; _Lap_, Lapinha; *_A_*, ancestral species.

Shared variation may be the result of gene flow or the incomplete sorting of ancestral polymorphism. The distinction between both possibilities is the main goal of several statistical tests [63] like the one implemented by IM through coalescent simulations [12]. Based on multilocus estimates of population migration rates showing nonzero peaks, our results strongly suggested that after separation, the two lineages have undergone gene flow in both directions for the sympatric and for the allopatric species pairs (Table 4, Figure 6C). Although the effective calculated number of gene migrants per generation indicates a bidirectional migration in both cases, gene flow is five to ten times higher in the sympatric species. In addition, the observed level of introgression is highly asymmetrical, with Sobral 2S receiving about seven times more migrants than Sobral 1S. The estimates of the mean time of migration events [13] indicate that most of them occurred between 0.13 MYA and 0.20 MYA, suggesting lower current levels of gene flow.

**Figure 7:**
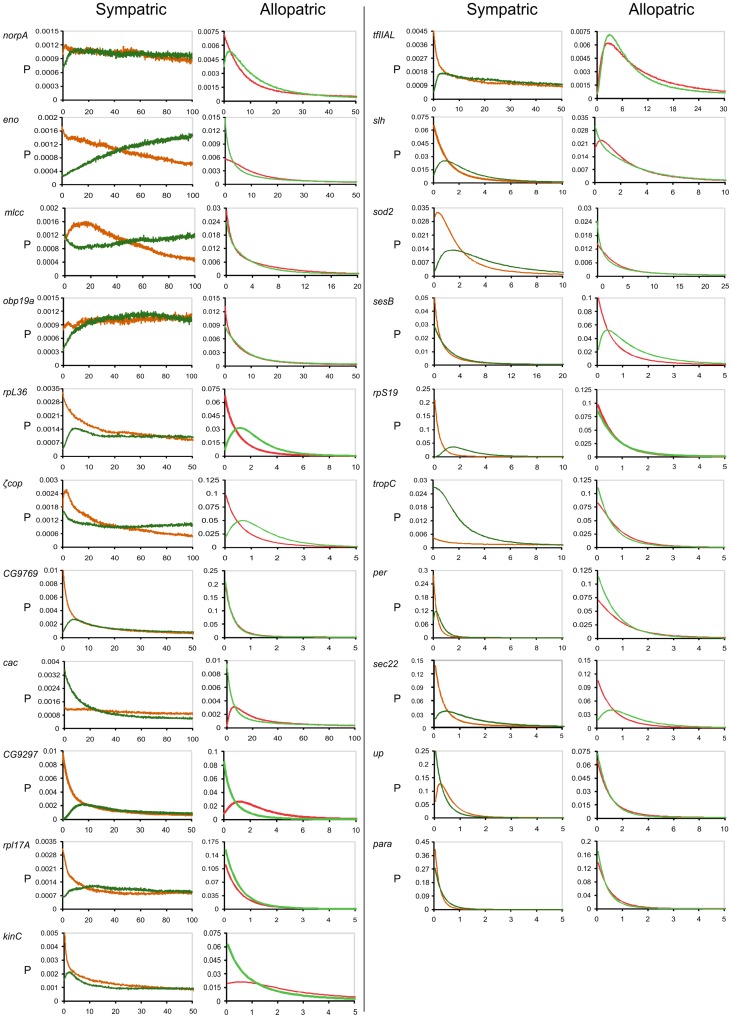
Distribution of marginal posterior probabilities of the migration rates at each locus. Probability densities (P) of migration estimates for 21 nuclear loci for two comparisons, sympatric - Sobral 1S (orange) *vs*. Sobral 2S (green) - and allopatric - Lapinha (red) *vs*. Pancas (light green).

We also estimated the per locus migration rates. Figure 7 shows the marginal posterior distributions for each gene. Migration at several loci was observed in both pairs of species. However, although sympatric and allopatric comparisons showed a similar number of loci that exhibited signals of gene flow, in general the allopatric comparison showed much lower levels of gene flow. In addition, most loci showed unidirectional gene flow and introgression from Sobral 1S to Sobral 2S. Weak divergence and extensive gene flow may result in a flat and diffuse posterior distribution [64], and this most likely explains the results observed for a number of loci in the case of the Sobral sympatric siblings.

## Discussion

The analysis of the divergence and gene flow at 21 nuclear loci in sympatric and allopatric Brazilian populations of *L. longipalpis s.l.* shows an intricate evolutionary history. The results suggest that, at least for the Brazilian species, introgressive hybridization has played a crucial role in the speciation process in this complex. In closely related species, this phenomenon seems to be common, and multilocus analyses have been useful in these cases [4], [5]. Overall, our results strongly suggest that introgression has played an active role in shaping the genomes of species in the *L. longipalpis* complex, as previously shown, for example, in *Anopheles*, *Heliconius* and *Drosophila* closely related species (e.g. [8], [22], [65]–[67].

The occurrence of sympatric species of the *L. longipalpis* complex in Sobral was strongly supported by our analysis, and this finding agrees with previous work [32]. Moreover, the high number of shared polymorphisms, some full shared haplotypes in a number of loci and the lack of fixed polymorphisms (except those previously reported for *para* [44] were the outstanding genetic traits observed in those two sibling species. The extremely variable levels of divergence among loci were the first clue that an evolutionary model of isolation with migration might fit our data [5], [14], [68].

The comparison between sympatric *vs.* allopatric populations provide further evidence for the occurrence of introgression between the sympatric species. If the sympatric pair alone had been analyzed, it might have been more difficult to determine whether the low divergence observed in some loci was merely a consequence of retention of ancestral polymorphisms or was the result of gene flow. However, as in some other studies (e.g., [23], [69], [70], the sympatric/allopatric comparison provided a clearer picture of the divergence process and indicated the occurrence of gene flow, which was confirmed by the IM analysis [12].

Interestingly, the allopatric sibling data also fit the isolation with migration model, which might reflect gene flow coming from populations of the same species subject to introgression in sympatry. The estimates of migration rates, time of divergence and mean *F*_ST_ between the allopatric sibling species, all satisfy the tentative threshold criteria for species diagnosis proposed by Hey and Pinho [71]. However, in the case of the sympatric species, the situation is a bit less clear with introgression affecting the mean *F*_ST_ and migration rates.

The observed level of introgression is highly asymmetrical with Sobral 2S receiving about seven times more migrants than Sobral 1S. Asymmetry in gene flow might be caused by differences in population density, and the gene flow direction is often predominantly from the most abundant species to the least [72]–[74]. In fact, our estimates of the *θ* population size parameters suggest that Sobral 1S is larger than Sobral 2S. The evidence for differential introgression among loci fits the mosaic model of speciation (e.g. [8], [22], [75], [76] in sympatric and allopatric species and can be used to identify genomic regions containing genes involved in speciation [77]. Some genes may have freely crossed the species boundaries; these genes include housekeeping and other genes that may not be associated with reproductive isolation or species-specific adaptation (e.g., *sod2* and *tfIIAL*) and were therefore not subjected to selective pressure against introgression [78]. Among the loci used in this study, three (*per*, *cac*, and *para*) are known to control courtship songs in *Drosophila* [79] and, interestingly, two (*per* and *para*) are among those with highest *F*_ST_ values. Future analysis of other song genes may reveal whether these genes tend to show higher levels of divergence and lower levels of gene flow between the *L. longipalpis* sibling species.

Assuming that the speciation process between the Burst/Cembrene and Pulse/Germacrene lineages occurred in allopatry (∼0.5 MYA), followed by secondary contact in localities such as Sobral, the evidence for introgression provided by the data suggests that, at first, the period of separation was not long enough to ensure the appearance of full reproductive isolation mechanisms. Following the secondary contact and a period of stronger hybridization and introgression, reinforcement of reproductive isolation might have promoted the evolution of more efficient mate discrimination, and other mechanisms of isolation could have taken place [67], [80]. Differences in male sex pheromones [35], copulation songs [39], locomotor activity rhythms [81] and life cycle traits [82] between Sobral 1S and 2S indicate that selection might be playing an active role in the divergence process of the two sibling species. In fact, there is evidence that the reproductive isolation between the sympatric Sobral siblings is stronger than between allopatric siblings of the *L. longipalpis* complex [29], [40], and gene flow might therefore be currently diminished.

In addition to Sobral, two other pairs of sympatric species in Jaíba (Bahia State) and Estrela de Alagoas (Alagoas State) localities were previously described in Brazil. In both cases, a Burst-type song and Cembrene-1 population, coexist in sympatry with a different Pulse-type song sibling [33]. Unlike Sobral, in Jaíba the two siblings differ in the type of diterpene isomers that they carry and in Estrela de Alagoas the siblings share the same type of pheromone [83]. Future work might reveal semipermeable species boundaries which would not necessarily involve the same genes, intensity and/or direction of gene flow.

Nevertheless, our results indicate that past gene flow has affected several areas of the *L. longipalpis s.l.* genome. In some cases, genomic regions with suppressed or reduced recombination, as a result of chromosomal rearrangements such as inversions, might have less introgression than colinear regions [84], [85]. Genes located in such regions might be involved in adaptive differences or reproductive divergence between siblings, and therefore be filtered by selection, while genes that are not linked to such regions might introgress more readily [8], [66]. In *L. longipalpis s.l.*, putative chromosomal inversions between some siblings have been identified [86], but future mapping experiments are needed to reveal which genome regions of this species complex have undergone more introgression than others. Furthermore, the knowledge of introgression patterns throughout the genome is important to understand whether loci related to vectorial capacity can influence the transmission dynamics of *Leishmania* parasites by the different *L*. *longipalpis* sibling species. This will be particularly interesting under an epidemiological point of view considering the potential for increased introgression caused by human-made changes to the environment [87] and the current trend of visceral leishmaniasis urbanization in Brazil [88].

## Supporting Information

Table S1 Chromosome positions of the 21 loci in *D. melanogaster* and *A. gambiae* and their biological functions and processes.
(DOC)

Table S2 List of primers used for 18 new markers of the 21 loci used in the multilocus analysis.
(DOC)

Table S3 Domains of the 21 loci and description of the non-recombining blocks constructed for the IM analyzes.
(DOC)

Table S4 Additional neutrality tests for 21 locus in four *L. longipalpis s.l.* populations of Brazil.
(DOC)

Table S5 Differentiation among siblings of *L. longipalpis* complex from Brazil.
(DOC)

Table S6 The average number of nucleotide substitutions per site among siblings of *L. longipalpis* species complex of Brazil, Dxy (Nei 1987).
(DOC)

Table S7 Polymorphism summary of the non-recombining blocks for the 21 loci in four sibling species of the *L. longipalpis* complex from Brazil.
(DOC)

Table S8 Differentiation in the non-recombining blocks between sympatric and allopatric species of the *L. longipalpis* complex from Brazil.
(DOC)
