# Supplementary material for: Multilocus Analysis of Divergence and Introgression in Sympatric and Allopatric Sibling Species of the Lutzomyia longipalpis Complex in Brazil
Source: PLoS Negl Trop Dis. 2013 Oct 17;7(10):e2495. doi: 10.1371/journal.pntd.0002495 (PMC3798421; doi:10.1371/journal.pntd.0002495)
Supplement: Table S1 — Chromosome positions of the 21 loci in D. melanogaster and A. gambiae and their biological functions and processes. (DOC) [file pntd.0002495.s001.doc]

**Supplementary table 1. Chromosome positions of the 21 loci in *D. melanogaster* and *A. gambiae* and their biological functions and processes.**

| Locus | *D. mel*^1^ | *A. gam*^2^ | Biological Function and Process |
| --- | --- | --- | --- |
| *CG9297* | *3R* | *2R* | GTP binding, GTPase activity, calcium ion binding. Calcium ion transport. |
| *CG9769* | *3R* | *2R* | Translation initiation factor activity. Autophagic cell death, salivary gland cell autophagic cell death, translational initiation. |
| *eno* | *2L* | *3R* | Magnesium ion binding, phosphopyruvate hydratase activity. Glycolysis. |
| *kinC* | *3R* | *2R* | ATP binding, calcium dependent protein kinase C activity, diacylglycerol binding, protein kinase C activity, protein serine/threonine kinase activity. Intracellular signal transduction, protein phosphorylation, response to ethanol. |
| *mlcc* | *x* | *3R* | ATPase activity, coupled, calcium ion binding, myosin binding, myosin heavy chain binding. |
| *norpA* | *x* | *2R* | Hydrolase activity. |
| *obp19a* | *x* | *2L* | Odorant binding. Olfactory behavior, response to pheromone, sensory perception of chemical stimulus, transport. |
| *rpL17A* | *2R* | *3R* | Protein binding, structural constituent of ribosome. Mitotic spindle elongation and organization, translation. |
| *rpL36* | *x* | *2R* | Structural constituent of ribosome. Translation. |
| *rpS19* | *x* | *3L* | Structural constituent of ribosome. Translation. |
| *sesB* | *x* | *2R* | ATP: ADP antiporter activity, binding, transmembrane transporter activity. ADP transport, determination of adult lifespan, flight behavior, locomotion, muscle cell homeostasis, neuron homeostasis, regulation of action potential, synaptic growth at neuromuscular junction, synaptic vesicle transport. |
| *slh* | *2L* | *3L* | SNARE binding. Intracellular protein transport, phagocytosis, engulfment, protein targeting, vesicle docking involved in exocytosis, vesicle-mediated transport, wing disc dorsal/ventral pattern formation. |
| *sec22* | *x* | *unknown* | SNAP receptor activity. Phagocytosis, engulfment, vesicle-mediated transport. |
| *sod2* | *2R* | *3L* | Antioxidant activity, metal ion binding, superoxide dismutase activity. Determination of adult lifespan, oxidation-reduction process, regulation of metabolic process, removal of superoxide radicals, superoxide metabolic process. |
| *tfIIAL* | *3R* | *2R* | General RNA polymerase II transcription factor activity. Regulation of transcription from RNA polymerase II promoter. |
| *tropC* | *3L* | *2L* | Calcium ion binding. |
| *up* | *x* | *2R* | Calcium ion binding, tropomyosin binding. Cellular calcium ion homeostasis, mesoderm development, mitochondrion organization, muscle cell homeostasis, myofibril assembly, sarcomere organization. |
| ζ*cop* | *3L* | *2L* | Phagocytosis, engulfment, regulation of lipid storage. |
| *cac* | *x* | *2L* | Calcium ion binding, voltage-gated calcium channel activity. Adult locomotor behavior, calcium ion transport, courtship behavior, detection of light stimulus involved in visual perception, epithelial fluid transport, exocytosis, male courtship behavior. |
| *para* | *x* | *2L* | Voltage-gated sodium channel activity. Male courtship behavior, response to DDT, pyrethroid, sodium ion transport, transmembrane transport. |
| *per* | *x* | *2R* | Protein binding, protein heterodimerization activity, signal transducer activity, transcription cofactor activity, transcription co-repressor activity. Age-dependent response to oxidative stress, circadian rhythm, copulation, courtship behavior, determination of adult lifespan, eclosion rhythm, locomotors rhythm. |

^1^, *D. melanogaster*; ^2^, *A. gambiae*. Available at <http://www.ncbi.nlm.nih.gov/> and <http://www.vectorbase.org/>.
