# Supplementary material for: Multilocus Analysis of Divergence and Introgression in Sympatric and Allopatric Sibling Species of the Lutzomyia longipalpis Complex in Brazil
Source: PLoS Negl Trop Dis. 2013 Oct 17;7(10):e2495. doi: 10.1371/journal.pntd.0002495 (PMC3798421; doi:10.1371/journal.pntd.0002495)
Supplement: Table S2 — List of primers used for 18 new markers of the 21 loci used in the multilocus analysis. (DOC) [file pntd.0002495.s002.doc]

**Supplementary table 2. List of primers for 18 new markers of the 21 loci used in the multilocus analysis.**

| Locus | Primer 5’ |  | Primer 3’ |  |
| --- | --- | --- | --- | --- |
| *CG9297* | 5LLCG9297 | CGATCAGGTGAAACCCGAAG | 3LLCG9297 | AGCGTGAGATGTTGGTTAGAG |
| *CG9769* | 5LLCG9769 | ATACGAACGGAGAAATGCAG | 3LLCG9769 | TCCACGGTAACATGCACAG |
| Enolase (*eno*) | 5LLeno | ATCCAGAAGATTGTTGCTCG | 3LLeno | CAAACAGCCAGAGATACACC |
| Protein C kinase 98E (*kinC*) | 5LLkinC98E | AGACGCGACACATGATGAC | 3LLkinC98E | AGTCCTGCTGTTCCTTCTC |
| Myosin light chain cytoplasmic (*mlcc*) | 5LLmlcc | AGCTGAATCCAGACGAGAG | 3LLmlcc | CCTCGTAGTTCACATTGCC |
| No receptor potential A (*norpA*) | 5LLnorpA | CATCCAAAGAAGTTGCCAATG | 3LLnorpA | TTTTTCCTTTTCCCTTCCCTG |
| Odorant-binding protein 19a (*obp19a*) | 5LLobp19a | CAACGAGATCATTGAGAGCG | 3LLobp19a | ATCCTCGACAGGCATCTAC |
| Ribosomal protein L17A (*rpL17A*) | 5LLrpL17A | TCAATTGCGCCGACAATAC | 3LLrpL17A | GCTGATCCTTTCATTTCGCC |
| Ribosomal protein L36 (*rpL36*) | 5LLrpL36 | GAATTTGCGGTACACAGGAG | 3LLrpL36 | TGTGTCAGGATGTTGGAGAG |
| Ribosomal protein S19a (*rpS19a*) | 5LLrp219 | CCAGGCGTTACAGTGAAAG | 3LLrp219 | TAGTGAGAGGGATGGACACC |
| Stress-sensitive B (*sesB*) | 5LLses-B | GTATTGTTGGGCTGTACCG | 3LLses-B | TGTAGATGGGTGTCTTCTTGG |
| SLY-1 homologous (*slh*) | 5LLslh | TCACCGCTCATTTCCATCC | 3LLslh | AGATCTTCCAGCTTCTGCC |
| VAMP-like protein (*sec22*) | 5LLsec22 | AGATGACAATGATTGCTCGG | 5LLsec22 | CAATGAAGGCATACGGTCTC |
| Superoxide dismutase 2 (Mn) (*sod2*) | 5LLsod2 | CATACCCTACCACAGCTTCC | 3LLsod2 | GAACAGCCACAGTGATTCC |
| Transcription factor IIA-L (*tfIIAL*) | 5LLtfIIAL | GATAATGATCCAGACGATGCC | 3LLtfIIAL | GAAAACATAGTCCTTCCCACC |
| Troponin C at 73F (*tropC*) | 5LLtropC | TCAGCCGTTCAACAAGAAG | 3LLtropC | TTCCAGATCCATCGGAGTC |
| Upheld (*up*) | 5LLup | GCAACAAGTCCAAAGAGCAG | 3LLup | TCATAGGAGCGGGTGTCAAC |
| ζCOP (ζ*cop*) | 5LLzetacop | GGATGCAGATCCTTCATCCG | 3LLzetacop | CGACCACTTCAGTTGTTCTC |
