# Supplementary material for: Multilocus Analysis of Divergence and Introgression in Sympatric and Allopatric Sibling Species of the Lutzomyia longipalpis Complex in Brazil
Source: PLoS Negl Trop Dis. 2013 Oct 17;7(10):e2495. doi: 10.1371/journal.pntd.0002495 (PMC3798421; doi:10.1371/journal.pntd.0002495)
Supplement: Table S3 — Domains of the 21 loci and description of the non-recombining blocks constructed for the IM analyzes. (DOC) [file pntd.0002495.s003.doc]

**Supplementary table 3. Domains of the 21 loci and description of non-recombining blocks constructed for the** IM analyzes.

| **Locus** | **Domains**^1^ | **Removed sites**^2^ | **NRB**^3^ |
| --- | --- | --- | --- |
| *CG9297* | E(1-221), I(222-329), E(330-474) | 235-240, 243, 247, 248, 262, 263, 321-325 | 173-295 |
| *CG9769* | E(1-30), I(31-96), E(97-243), I(244-300), E(301-378) | 286-290 | 1-378 |
| *eno* | E(1-245) | - | 1-222 |
| *kinC* | E(1-68), I(69-494), E(495-664) | 235, 413, 450-453, 463-465 | 137-663 |
| *mlcc* | E(1-217) | - | 1-217 |
| *norpA* | E(1-104) | - | 1-94 |
| *obp19a* | E(1-164) | - | 29-136 |
| *rpL17A* | E(1-130), I(131-200), E(201-302) | 143-145, 167, 168, 174, 177-180 | 1-195 |
| *rpL36* | E(1-41), I(42-339), E(340-450) | - | 216-333 |
| *rpS19* | E(1-49), I(50-116), E(117-300) | 110, 111 | 1-225 |
| *sesB* | E(1-89) | - | 1-89 |
| *slh* | E(1-34), I(35-109), E(110-258) | 77-81 | 56-141 |
| *sec22* | E(1-48), I(49-119), E(120-229), I(230-294), E(295-429) | 256, 290 | 60-424 |
| *sod2* | E(1-310) | - | 1-310 |
| *tfIIAL* | E(1-114), I(115-320), E(321-377) | 178-180, 217-220, | 133-264 |
| *tropC* | E(1-37), I(38-265), E(266-482) | 113-115, 161-163, 170, 171, 222, 247-249 | 1-380 |
| *up* | E(1-109), I(110-198), E(199-291), I(292-368), E(369-404) | 172, 177, 187-189 | 156-404 |
| *ζcop* | E(1-62), I(63-370), E(371-386) | 132, 133, 141-143, 184, 185, 191-194, 212, 225, 226, 258-260, 293, 294, 311, 344 | 47-386 |
| *cac* | I(1-109) | 17-26, 43, 57-59, 61-71, 78 | 1-85 |
| *para* | E(1-120), I(121-334), E(335-377) | 182, 183, 262, 281 | 20-283 |
| *per* | E(1-81), I(82-135), E(136-266) | - | 20-117 |

^1^, Exon (E) and Intron (I) regions for whole fragment alignment; ^2^, Sites with indels or ambiguous alignment removed during non-recombining block (NBR) construction; ^3^, Fragment positions of the NBR used in the IM analyzes.
