# Supplementary material for: Multilocus Analysis of Divergence and Introgression in Sympatric and Allopatric Sibling Species of the Lutzomyia longipalpis Complex in Brazil
Source: PLoS Negl Trop Dis. 2013 Oct 17;7(10):e2495. doi: 10.1371/journal.pntd.0002495 (PMC3798421; doi:10.1371/journal.pntd.0002495)
Supplement: Table S4 — Additional neutrality tests for 21 locus in four L. longipalpis s.l. populations of Brazil. (DOC) [file pntd.0002495.s004.doc]

**Table S4. Additional neutrality tests for 21 locus in four *L. longipalpis s.l.* populations of Brazil**

|  | Sobral 1S | | Sobral 2S | | Lapinha | | Pancas | |
| --- | --- | --- | --- | --- | --- | --- | --- | --- |
| locus | *F*_s_ | *R*^2^ | *F*_s_ | *R*^2^ | *F*_s_ | *R*^2^ | *F*_s_ | *R*^2^ |
| *CG9297* | -1.485 | 0.142 | -9.693* | 0.107 | -4.083 | 0.140 | -2.632 | 0.066 |
| *CG9769* | 0.508 | 0.125 | -3.339 | 0.077 | -0.879 | 0.218 | 0.055 | 0.123 |
| *eno* | -3.617* | 0.079 | -10.094* | 0.049* | -0.198 | 0.134 | -10.094* | 0.087 |
| *kinC* | -4.947 | 0.118 | -3.839 | 0.093 | -4.857 | 0.106 | -3.461 | 0.087 |
| *mlcc* | -6.180* | 0.056* | -5.702* | 0.062* | -4.412* | 0.117 | -1.531 | 0.108 |
| *norpA* | -2.972 | 0.121 | -4.742 | 0.143 | -4.141 | 0.085 | -4.020 | 0.125 |
| *obp19a* | -8.463 | 0.144 | -6.382 | 0.145 | -1.098 | 0.163 | -3.727 | 0.136 |
| *rpL17A* | -0.310 | 0.135 | -4.058 | 0.085 | 0.963 | 0.139 | 0.535 | 0.214 |
| *rpL36* | -4.161 | 0.099 | -2.718 | 0.117 | 2.675 | 0.165 | 0.836 | 0.120 |
| *rpS19* | -10.639* | 0.109 | -3.930 | 0.093 | -4.262 | 0.133 | -0.888 | 0.126 |
| *sesB* | -0.592 | 0.127 | 1.619 | 0.259 | 0.000* | 0.204 | -0.918 | 0.139 |
| *slh* | -8.166* | 0.153 | -7.655 | 0.081 | -1.171 | 0.106 | -1.915 | 0.133 |
| *sec22* | -4.754 | 0.084 | -0.658 | 0.127 | -2.662 | 0.100 | 3.477 | 0.143 |
| *sod2* | -0.966 | 0.115 | -4.739 | 0.074 | -3.383 | 0.157 | -1.963 | 0.121 |
| *tfIIAL* | -3.334 | 0.136 | -1.645 | 0.142 | -1.548 | 0.102 | 0.221 | 0.113 |
| *tropC* | -8.031* | 0.059* | -8.140* | 0.067 | -1.585 | 0.134 | -6.696* | 0.066 |
| *up* | -5.795 | 0.088 | -2.341 | 0.094 | -1.553 | 0.141 | 1.043 | 0.157 |
| *ζcop* | -3.476 | 0.073 | -0.089 | 0.102 | -1.751 | 0.102 | -0.238 | 0.114 |
| *cac* | -7.046* | 0.076 | -0.022 | 0.213 | -2.055 | 0.169 | 1.418 | 0.182 |
| *para* | -6.987* | 0.056* | -6.084 | 0.086 | 0.204 | 0.167 | -3.860 | 0.080 |
| *per* | -8.970* | 0.107 | -8.910* | 0.091 | -1.317 | 0.112 | -9.239* | 0.092 |

*F*_s_ = Fu´s *F*_s_ and *R*^2^ = Ramos Onsin´s statistics.

*, Significant values after Bonferroni´s correction (α > 0.0024).
