# Supplementary material for: Multilocus Analysis of Divergence and Introgression in Sympatric and Allopatric Sibling Species of the Lutzomyia longipalpis Complex in Brazil
Source: PLoS Negl Trop Dis. 2013 Oct 17;7(10):e2495. doi: 10.1371/journal.pntd.0002495 (PMC3798421; doi:10.1371/journal.pntd.0002495)
Supplement: Table S5 — Differentiation among siblings of L. longipalpis complex from Brazil. (DOC) [file pntd.0002495.s005.doc]

|  | Sobral 2S *vs* Pancas | | | Lapinha *vs* Sobral 1S | | | Sobral 1S *vs* Pancas | | | Sobral 2S *vs* Lapinha | | |
| --- | --- | --- | --- | --- | --- | --- | --- | --- | --- | --- | --- | --- |
| locus | F*_st_* | *Ss* | *Sf* | F*_st_* | *Ss* | *Sf* | F*_st_* | *Ss* | *Sf* | F*_st_* | *Ss* | *Sf* |
| *CG9297* | 0.159*** | 17 | 0 | 0.237*** | 18 | 0 | 0.281*** | 13 | 0 | 0.336*** | 17 | 0 |
| *CG9769* | 0.065** | 2 | 0 | 0.894*** | 0 | 2 | 0.000 ^ns^ | 2 | 0 | 0.920*** | 0 | 8 |
| *eno* | 0.000^ns^ | 2 | 0 | 0.138*** | 0 | 0 | 0.006 ^ns^ | 1 | 0 | 0.111*** | 0 | 0 |
| *kinC* | 0.093*** | 10 | 0 | 0.073* | 10 | 0 | 0.188*** | 11 | 0 | 0.307*** | 10 | 0 |
| *mlcc* | 0.240*** | 1 | 0 | 0.068** | 2 | 0 | 0.218*** | 1 | 0 | 0.105*** | 1 | 0 |
| *norpA* | 0.082** | 5 | 0 | 0.073** | 6 | 0 | 0.024 ^ns^ | 5 | 0 | 0.075* | 4 | 0 |
| *obp19a* | 0.051* | 10 | 0 | 0.081*** | 11 | 0 | 0.055 ^ns^ | 9 | 0 | 0.144*** | 10 | 0 |
| *rpL17A* | 0.179*** | 1 | 0 | 0.350*** | 4 | 0 | 0.430*** | 1 | 0 | 0.443*** | 5 | 0 |
| *rpL36* | 0.124*** | 18 | 0 | 0.062* | 17 | 0 | 0.179*** | 12 | 0 | 0.108*** | 14 | 0 |
| *rpS19* | 0.120* | 8 | 0 | 0.004 ^ns^ | 11 | 0 | 0.481*** | 2 | 0 | 0.356*** | 9 | 0 |
| *sesB* | 0.245* | 1 | 0 | 0.109* | 0 | 0 | 0.666*** | 0 | 0 | 0.440*** | 0 | 0 |
| *slh* | 0.301*** | 4 | 0 | 0.215*** | 8 | 0 | 0.370*** | 2 | 0 | 0.378*** | 8 | 0 |
| *sec22* | 0.290*** | 14 | 0 | 0.077** | 7 | 0 | 0.376*** | 5 | 0 | 0.559*** | 2 | 0 |
| *sod2* | 0.306*** | 2 | 0 | 0.232*** | 1 | 0 | 0.246*** | 2 | 0 | 0.444*** | 1 | 0 |
| *tfIIA-L* | 0.195*** | 18 | 0 | 0.029 ^ns^ | 16 | 0 | 0.298*** | 14 | 0 | 0.212*** | 16 | 0 |
| *tropC* | 0.130*** | 2 | 0 | 0.111*** | 1 | 0 | 0.438*** | 3 | 0 | 0.605*** | 0 | 0 |
| *up* | 0.260*** | 2 | 0 | 0.282*** | 6 | 0 | 0.533*** | 0 | 0 | 0.437*** | 3 | 0 |
| *ζcop* | 0.292*** | 3 | 0 | 0.284*** | 1 | 0 | 0.270*** | 4 | 0 | 0.356*** | 1 | 0 |
| *cac* | 0.000 ^ns^ | 6 | 0 | 0.122** | 6 | 0 | 0.019 ^ns^ | 4 | 0 | 0.143* | 4 | 0 |
| *para* | 0.077* | 3 | 0 | 0.137** | 1 | 0 | 0.761*** | 3 | 1 | 0.825*** | 0 | 4 |
| *per* | 0.019 ^ns^ | 12 | 0 | 0.124*** | 14 | 0 | 0.436*** | 3 | 0 | 0.434*** | 6 | 0 |

**Supplementary table 5. Differentiation among siblings of *L. longipalpis* complex from Brazil.**

*F*_ST_, pairwise fixation index. Significance evaluated with 1000 permutations; ***, significant at P < 0.001; **, significant at P < 0.01; *, significant at P < 0.05; ^ns^, non-significant P > 0.05. *Ss,* shared sites; *Sf,* fixed sites*.*
