# Supplementary material for: Multilocus Analysis of Divergence and Introgression in Sympatric and Allopatric Sibling Species of the Lutzomyia longipalpis Complex in Brazil
Source: PLoS Negl Trop Dis. 2013 Oct 17;7(10):e2495. doi: 10.1371/journal.pntd.0002495 (PMC3798421; doi:10.1371/journal.pntd.0002495)
Supplement: Table S6 — The average number of nucleotide substitutions per site among siblings of L. longipalpis species complex of Brazil, Dxy (Nei 1987). (DOC) [file pntd.0002495.s006.doc]

**Supplementary table 6. The average number of nucleotide substitutions per site among siblings of *L. longipalpis species* complex of Brazil, Dxy (Nei 1987)**

| Comparisons | Sympatric | Allopatric | | | | |
| --- | --- | --- | --- | --- | --- | --- |
| locus | Sobral 1S *vs* Sobral 2S | Lapinha *vs* Pancas | Sobral 2S *vs* Pancas | Sobral 1S *vs* Pancas | Sobral 2S *vs* Lapinha | Sobral 1S *vs* Lapinha |
| *CG9297* | 0.024 | 0.036 | 0.017 | 0.023 | 0.036 | 0.034 |
| *CG9769* | 0.004 | 0.023 | 0.003 | 0.003 | 0.024 | 0.023 |
| *eno* | 0.002 | 0.003 | 0.003 | 0.002 | 0.004 | 0.003 |
| *kinC* | 0.012 | 0.016 | 0.011 | 0.012 | 0.016 | 0.012 |
| *mlcc* | 0.006 | 0.010 | 0.008 | 0.009 | 0.007 | 0.008 |
| *norpA* | 0.017 | 0.024 | 0.021 | 0.023 | 0.017 | 0.020 |
| *obp19a* | 0.031 | 0.032 | 0.032 | 0.030 | 0.035 | 0.030 |
| *rpL17A* | 0.016 | 0.018 | 0.011 | 0.013 | 0.018 | 0.013 |
| *rpL36* | 0.029 | 0.025 | 0.026 | 0.025 | 0.029 | 0.024 |
| *rpS19* | 0.027 | 0.026 | 0.016 | 0.025 | 0.027 | 0.016 |
| *sesB* | 0.008 | 0.010 | 0.007 | 0.014 | 0.005 | 0.002 |
| *slh* | 0.027 | 0.030 | 0.023 | 0.033 | 0.023 | 0.024 |
| *sec22* | 0.026 | 0.025 | 0.025 | 0.026 | 0.025 | 0.010 |
| *sod2* | 0.011 | 0.007 | 0.009 | 0.008 | 0.012 | 0.008 |
| *tfIIA-L* | 0.030 | 0.030 | 0.031 | 0.028 | 0.032 | 0.021 |
| *tropC* | 0.006 | 0.005 | 0.004 | 0.005 | 0.006 | 0.002 |
| *up* | 0.018 | 0.017 | 0.010 | 0.020 | 0.015 | 0.015 |
| *ζcop* | 0.006 | 0.008 | 0.009 | 0.007 | 0.008 | 0.006 |
| *cac* | 0.029 | 0.053 | 0.026 | 0.027 | 0.054 | 0.053 |
| *para* | 0.017 | 0.017 | 0.005 | 0.018 | 0.016 | 0.003 |
| *per* | 0.034 | 0.030 | 0.016 | 0.033 | 0.031 | 0.024 |
