# Supplementary material for: Multilocus Analysis of Divergence and Introgression in Sympatric and Allopatric Sibling Species of the Lutzomyia longipalpis Complex in Brazil
Source: PLoS Negl Trop Dis. 2013 Oct 17;7(10):e2495. doi: 10.1371/journal.pntd.0002495 (PMC3798421; doi:10.1371/journal.pntd.0002495)
Supplement: Table S7 — Polymorphism summary of the non-recombining blocks for the 21 loci in four sibling species of the L. longipalpis complex from Brazil. (DOC) [file pntd.0002495.s007.doc]

**Supplementary table 7. Polymorphism summary of non-recombining blocks for the 21 loci in four siblings of the *L. longipalpis* complex from Brazil.**

|  |  | N | | | | S | | | | π | | | | *Ө* | | | |
| --- | --- | --- | --- | --- | --- | --- | --- | --- | --- | --- | --- | --- | --- | --- | --- | --- | --- |
| Locus | Length | S1S | S2S | Lap | Pan | S1S | S2S | Lap | Pan | S1S | S2S | Lap | Pan | S1S | S2S | Lap | Pan |
| *CG9297* | 117 | 29 | 23 | 11 | 23 | 11 | 14 | 14 | 6 | 0.024 | 0.024 | 0.055 | 0.007 | 0.024 | 0.032 | 0.042 | 0.014 |
| *CG9769* | 378 | 24 | 24 | 19 | 14 | 12 | 8 | 1 | 3 | 0.005 | 0.035 | 0.000 | 0.002 | 0.009 | 0.006 | 0.001 | 0.003 |
| *eno* | 222 | 28 | 32 | 29 | 30 | 3 | 8 | 3 | 4 | 0.001 | 0.003 | 0.004 | 0.001 | 0.004 | 0.009 | 0.003 | 0.005 |
| *kinC* | 508 | 15 | 20 | 21 | 18 | 10 | 12 | 13 | 9 | 0.005 | 0.004 | 0.005 | 0.004 | 0.006 | 0.007 | 0.007 | 0.005 |
| *mlcc* | 217 | 30 | 28 | 15 | 24 | 12 | 10 | 7 | 7 | 0.005 | 0.006 | 0.008 | 0.007 | 0.012 | 0.014 | 0.010 | 0.009 |
| *norpA* | 93 | 31 | 28 | 37 | 18 | 7 | 4 | 8 | 6 | 0.021 | 0.014 | 0.018 | 0.017 | 0.019 | 0.011 | 0.021 | 0.019 |
| *obp19a* | 108 | 25 | 26 | 24 | 17 | 5 | 7 | 6 | 5 | 0.012 | 0.017 | 0.016 | 0.011 | 0.012 | 0.017 | 0.015 | 0.014 |
| *rpL17A* | 178 | 28 | 27 | 23 | 14 | 9 | 11 | 4 | 2 | 0.016 | 0.014 | 0.007 | 0.005 | 0.013 | 0.016 | 0.006 | 0.004 |
| *rpL36* | 116 | 18 | 12 | 21 | 23 | 16 | 17 | 9 | 12 | 0.033 | 0.044 | 0.027 | 0.023 | 0.04 | 0.049 | 0.022 | 0.028 |
| *rpS19* | 222 | 24 | 28 | 17 | 9 | 10 | 14 | 5 | 5 | 0.01 | 0.019 | 0.006 | 0.007 | 0.012 | 0.016 | 0.007 | 0.008 |
| *sesB* | 89 | 23 | 26 | 21 | 15 | 2 | 1 | 0 | 2 | 0.004 | 0.006 | 0.000 | 0.004 | 0.006 | 0.003 | 0.000 | 0.007 |
| *slh* | 77 | 21 | 27 | 19 | 16 | 10 | 9 | 3 | 5 | 0.037 | 0.023 | 0.012 | 0.021 | 0.036 | 0.03 | 0.012 | 0.021 |
| *sec22* | 364 | 13 | 11 | 21 | 12 | 16 | 14 | 4 | 12 | 0.009 | 0.009 | 0.004 | 0.015 | 0.014 | 0.013 | 0.003 | 0.012 |
| *sod2* | 310 | 26 | 26 | 18 | 14 | 10 | 14 | 4 | 5 | 0.008 | 0.008 | 0.005 | 0.004 | 0.009 | 0.012 | 0.004 | 0.005 |
| *tfIIAL* | 128 | 9 | 14 | 16 | 34 | 9 | 20 | 14 | 15 | 0.028 | 0.051 | 0.028 | 0.03 | 0.026 | 0.049 | 0.033 | 0.029 |
| *tropC* | 373 | 23 | 26 | 29 | 31 | 9 | 10 | 3 | 8 | 0.003 | 0.004 | 0.001 | 0.003 | 0.007 | 0.007 | 0.002 | 0.005 |
| *up* | 245 | 19 | 20 | 14 | 15 | 14 | 6 | 8 | 6 | 0.01 | 0.006 | 0.011 | 0.008 | 0.016 | 0.007 | 0.01 | 0.008 |
| *ζcop* | 323 | 26 | 15 | 26 | 21 | 11 | 9 | 4 | 7 | 0.005 | 0.006 | 0.004 | 0.006 | 0.009 | 0.009 | 0.003 | 0.006 |
| *cac* | 59 | 22 | 22 | 16 | 26 | 7 | 4 | 11 | 3 | 0.019 | 0.029 | 0.066 | 0.021 | 0.033 | 0.019 | 0.056 | 0.013 |
| *para* | 259 | 27 | 20 | 21 | 29 | 11 | 4 | 2 | 4 | 0.005 | 0.002 | 0.002 | 0.003 | 0.011 | 0.004 | 0.002 | 0.004 |
| *per* | 98 | 16 | 21 | 16 | 18 | 7 | 9 | 7 | 6 | 0.02 | 0.013 | 0.013 | 0.012 | 0.022 | 0.026 | 0.022 | 0.018 |

N, number of sequences; S, segregating sites; π, average number of pair-wise differences; *Ө*, neutral parameter, based on the number of segregating sites.
