# Supplementary material for: Multilocus Analysis of Divergence and Introgression in Sympatric and Allopatric Sibling Species of the Lutzomyia longipalpis Complex in Brazil
Source: PLoS Negl Trop Dis. 2013 Oct 17;7(10):e2495. doi: 10.1371/journal.pntd.0002495 (PMC3798421; doi:10.1371/journal.pntd.0002495)
Supplement: Table S8 — Differentiation in the non-recombining blocks between sympatric and allopatric species of the L. longipalpis complex from Brazil. (DOC) [file pntd.0002495.s008.doc]

**Supplementary table 8. Differentiation in the non-recombining blocks between sympatric and allopatric species of the *L. longipalpis* complex from Brazil.**

|  | Sympatric | | |  |  | Allopatric | | |  |  |
| --- | --- | --- | --- | --- | --- | --- | --- | --- | --- | --- |
| Locus | *F*_ST_ | *Ss* | *Sf* | *S*_S1S_ | *S*_S2S_ | *F*_ST_ | *Ss* | *Sf* | *S*_Lap_ | *S*_Pan_ |
| *CG9297* | 0.071* | 8 | 0 | 3 | 6 | 0.307*** | 1 | 0 | 13 | 5 |
| *CG9769* | 0.059* | 3 | 0 | 12 | 8 | 0.953*** | 0 | 8 | 1 | 3 |
| *eno* | 0.010 ^ns^ | 1 | 0 | 2 | 7 | 0.144*** | 0 | 0 | 3 | 4 |
| *kinC* | 0.244*** | 3 | 0 | 7 | 9 | 0.331*** | 0 | 0 | 13 | 9 |
| *mlcc* | 0.021 ^ns^ | 3 | 0 | 12 | 10 | 0.250*** | 0 | 0 | 7 | 7 |
| *norpA* | 0,000 ^ns^ | 5 | 0 | 3 | 0 | 0.152*** | 2 | 0 | 6 | 5 |
| *obp19a* | 0.049 ^ns^ | 4 | 0 | 1 | 3 | 0.1423** | 1 | 0 | 5 | 4 |
| *rpL17A* | 0.049 ^ns^ | 7 | 0 | 2 | 4 | 0.739*** | 0 | 2 | 4 | 2 |
| *rpL36* | 0.061* | 8 | 0 | 8 | 9 | 0.269*** | 3 | 0 | 6 | 9 |
| *rpS19* | 0.436*** | 6 | 0 | 4 | 8 | 0.747*** | 0 | 4 | 5 | 5 |
| *sesB* | 0.334*** | 0 | 0 | 2 | 1 | 0.796*** | 0 | 0 | 0 | 2 |
| *slh* | 0.122*** | 3 | 0 | 7 | 6 | 0.283*** | 0 | 0 | 3 | 6 |
| *sec22* | 0.690*** | 0 | 0 | 16 | 14 | 0.634*** | 0 | 2 | 4 | 16 |
| *sod2* | 0.274*** | 6 | 0 | 4 | 8 | 0.359*** | 0 | 0 | 4 | 5 |
| *tfIIAL* | 0.203*** | 8 | 0 | 1 | 12 | 0.504*** | 13 | 0 | 1 | 2 |
| *tropC* | 0.364*** | 2 | 0 | 7 | 8 | 0.633*** | 0 | 1 | 3 | 8 |
| *up* | 0.539*** | 1 | 0 | 13 | 5 | 0.491*** | 0 | 1 | 8 | 6 |
| *ζcop* | 0.073* | 4 | 0 | 7 | 5 | 0.476*** | 0 | 0 | 4 | 7 |
| *cac* | 0.078^ns^ | 3 | 0 | 4 | 1 | 0.071 ^ns^ | 3 | 0 | 8 | 0 |
| *para* | 0.803*** | 0 | 2 | 12 | 4 | 0.853*** | 0 | 3 | 2 | 4 |
| *per* | 0.556*** | 0 | 0 | 7 | 9 | 0.659*** | 0 | 1 | 7 | 6 |

*F*_ST_, pairwise fixation index. Significance evaluated with 1000 permutations; *** significant at P < 0.001; ** significant at P < 0.01; * significant at P < 0.05; ^ns^ non-significant P > 0.05. *Ss,* shared sites; *Sf,* fixed sites; *S*, number of polymorphic sites*.* S2S, Sobral 2S; S1S, Sobral 1S; Pan, Pancas; Lap, Lapinha.
